# Diagnostic Accuracy of Next-Generation Sequencing: Prevalence of HIV-1 Drug Resistance and Associated Factors Among Adults on Integrase Inhibitors with Virologic Failure

**DOI:** 10.3390/v17121596

**Published:** 2025-12-09

**Authors:** Sandra Lunkuse, Ronald Kiiza, Alfred Ssekagiri, Maria Nannyonjo, Nathan Ntenkaire, Faridah Nassolo, Hamida Suubi Namagembe, Faizo Kiberu, Danstan Kabuuka, Irene Andia, Joan Nakayaga Kalyango, Pauline Byakika Kibwika, Nicholas Bbosa, Pontiano Kaleebu, Deogratius Ssemwanga

**Affiliations:** 1 Sequencing Platform, Medical Research Council (MRC)/UVRI & London School of Hygiene and Tropical Medicine (LSHTM), Uganda Research Unit, Entebbe P.O Box 49, Uganda; 2 Clinical Epidemiology Unit, College of Health Science, Makerere University, Kampala P.O. Box 7072, Uganda; 3 Department of General Virology, Uganda Virus Research Institute (UVRI), Entebbe P.O Box 49, Uganda

**Keywords:** next-generation sequencing, virologic failure, high-frequency variants, minority variants, HIV-1 drug resistance, integrase strand transfer inhibitors

## Abstract

Emerging evidence indicates a high rate (>10%) of drug resistance (DR) associated with integrase strand transfer inhibitors (INSTIs) in developed countries, although there is limited information on DR during INSTI treatment in Uganda. With the increased use of INSTIs as standard first-line treatment, monitoring for DR using next-generation sequencing (NGS) has become essential. NGS can detect the lower-frequency variants that may be missed by traditional Sanger sequencing (SS). This study evaluates the diagnostic accuracy of next-generation sequencing (NGS) compared to Sanger sequencing for detecting HIV-1 INSTI resistance mutations and estimates the prevalence and factors associated with drug resistance among adults with virologic failure on INSTI-based regimens in Uganda. Utilizing the Illumina MiSeq platform for NGS, data was analyzed using STATA V.18 and a logistic regression model at 5% level of significance. This study demonstrates that NGS achieved 100% sensitivity, specificity, positive predictive value, negative predictive value, and overall accuracy in detecting major mutations. NGS identified INSTI DRMs in 4% of adults at a ≥20% threshold and was able to detect both high- and low-abundance variants, which could have important implications for clinical outcomes. This study emphasizes the need for HIVDR testing before antiretroviral therapy (ART) initiation, given the increasing use of INSTIs. We recommend that healthcare providers adopt more sensitive diagnostics such as NGS and use detailed resistance profiles to tailor antiretroviral therapies. This approach is critical for effectively managing and preventing drug-resistant HIV strains.

## 1. Introduction

Despite efforts to combat HIV, it remains a significant public health issue, with approximately 40.8 million individuals living with the virus worldwide by the end of 2024 [1,2]. Sub-Saharan Africa bears most of this global burden, accounting for two-thirds of cases [1]. At present, the occurrence of HIV among individuals aged 15–49 in Uganda stands at 5.5%, representing a slight decrease from the 6.0% observed in the Uganda population-based HIV impact assessment (UPHIA) 2016-17 [3]. The Joint United Nations Programme on HIV/AIDS (UNAIDS) established the 95-95-95 objectives for 2030, which require 95% of people living with HIV to be aware of their status, 95% of those with known status to be on treatment, and 95% of the treated to achieve virological suppression [4].

Viral load suppression is a critical indicator of good treatment outcomes and the absence of drug resistance to active treatments [5]. To address the issue of drug resistance, the World Health Organization (WHO) advised countries with pre-treatment non-nucleoside reverse-transcriptase inhibitor (NNRTI) resistance exceeding 10% to transition to Integrase Strand Transfer Inhibitors (INSTIs). Several low- and middle-income countries (LMICs), including Uganda, have adopted dolutegravir and raltegravir as the preferred initial treatment options for adults with HIV [6]. Uganda approved and introduced Lenacapavir and Cabotegravir (CAB-LA): highly effective long-acting injectables that provide protection against HIV [7]. By September 2021, 78% of those on antiretroviral therapy (ART) were receiving the TDF/3TC/DTG (TLD) regimen. In most PEPFAR-supported countries, over 80% of people with HIV on ART were on TLD [8]. Despite the high efficacy of INSTIs, resistance to these regimens among adults is possible, leading to virological treatment failure [9].

The WHO recommends HIV drug-resistance testing at entry into care for individuals initiating HIV antiretroviral therapy (ART) to guide the selection of the initial regimen, as well as during treatment to inform the selection of active drugs when switching ARV regimens in antiretroviral therapy (ART) experienced people experiencing virologic failure [1]. However, such testing is not performed in resource-limited settings like Uganda; hence, persons switched to INSTIs are not assessed for resistance [10]. Presently, the WHO does not propose specific thresholds for switching regimens for virological failures on DTG-based ART. Studies in other settings have shown resistance to INSTIs at magnitudes >10% [11,12,13], hence threatening the effectiveness of DTG and other INSTIs [14].

Drug resistance to INSTI may result from undocumented prior exposure to the drug, the transmission of resistant strains during the initial infection, or poor treatment adherence, among other factors. DRMs raise viral load, resulting in a decrease in CD4 cell count (white blood cells). A low CD4 cell count below 200 cells/µL weakens an individual’s immunity, increasing the risk of severe infections, some cancers, other long-term complications, progression to AIDS, death, and a higher probability of HIV transmission [5].

Studies have shown that both next-generation sequencing (NGS) and Sanger sequencing are utilized for HIV drug resistance (HIVDR) testing in Uganda, although Sanger sequencing is more prevalent [10,13]. Sanger sequencing detects major mutations but is limited by the non-detection of minority variants with prevalence below 20% in the viral population [15]. Minority variants are associated with an increase in the risk of treatment failure to the administered antiretroviral drugs, which may have significant clinical implications [16]. In addition to higher throughput and lower cost when analyzing batched samples, NGS could be a viable alternative to Sanger sequencing. Nevertheless, its clinical usefulness has yet to be extensively examined in resource-limited countries. The burden of drug resistance among people on INSTIs has not been documented in Uganda. Therefore, this study aimed to validate the use of NGS in the detection of HIVDR and evaluate the prevalence and factors associated with HIV drug resistance among adults experiencing virologic failure in Uganda.

## 2. Materials and Methods

### 2.1. Study Design and Population

We conducted a cross-sectional study on archived samples from adults receiving INSTIs based ART regimen in Uganda from 2022 to 2023. Samples from a cross-sectional survey of 857 PLHIV who were virologically failing were used to determine the prevalence of HIVDR using the Sanger sequencing platform.

Participants eligible for the study were at least 18 years of age, living with HIV on any INSTIs for at least a year with VF, whose samples were received at the Uganda Virus Research Institute for drug-resistance testing from January 2022 to January 2023 and did not have previous exposure to INSTIs before the current regimen.

#### Data Collection

Systematic random sampling was used to select 253 participants on INSTIs for at least one year with virologic failure from the HIVDR database. Data abstraction forms were used to obtain socio-demographic, clinical, and medical history data from reviewing the HIV-drug-resistance database. For data regarding the presence of INSTI-drug-resistance mutation, the dependent variable which was a binary outcome was obtained through extraction, amplification, visualizing and sequencing as detailed below.

### 2.2. Laboratory Methods

#### 2.2.1. RNA Extraction, Amplification, and Visualization

Genotyping of the integrase region was performed using a validated in-house method in the WHO-designated laboratory. Ribonucleic acid (RNA) was extracted from 500 µL of plasma using the NucliSENS easyMAG (Biomeriux). Identification codes of the samples to be processed were entered using a barcode reader after logging into the software. The extraction protocol (on board), sample volume (500 µL), and elution volume (40 µL) were selected on the instrument, and sample identification codes were assigned to a run. The reagent bottles containing lysis buffer, extraction buffers 1, 2, and 3, along with sample strips and aspirator disposables, were loaded onto the instrument and scanned using a barcode reader. Then, 500 µL of the plasma sample was transferred to the corresponding sample strip as entered in the software, initiating the lysis procedure, which ran for 10 min. A pre-mix consisting of 550 µL of internal control (RNA or DNA) and 550 µL of silica solution was vortexed to ensure homogeneity and then transferred to a trough. From the trough, 100 µL of the premix was transferred to the sample strips containing the lysed samples, discarding the pipette tips after each transfer. The mixture was properly homogenized by pipetting up and down. The run was resumed to perform incubation, washing, elution, and RNA separation from the elution buffer for 45 min. The concentrated nucleic acid (eluate) 40 µL was pipetted off from the sample strip and transferred to a clean labeled 1.5 mL Eppendorf tube for storage at −80 °C.

From the eluate, 10 µL was used for amplification of the integrase region using the HIV-1 genotyping kit with integrase (CDC Atlanta), as suggested by the manufacture’s protocol. RT-PCR was performed under specific thermocycling conditions using the Gene Amp PCR system 9700 thermocycler.

Nested polymerase chain reaction (PCR) was performed using 2 µL of the primary PCR product, the nested master mix from the HIV-1 genotyping kit with integrase and platinum Taq HIFI enzyme. Nested PCR was performed under specific thermocycling conditions using the Gene Amp PCR system 9700 thermocycler. The correct size of the amplified product was visualized using agarose gel electrophoresis using a 1% agarose gel stained with red safe.

#### 2.2.2. Sanger Sequencing

A 5′ region of the pol gene, specifically the HIV-1 integrase region consisting of approximately 900 base pairs, was sequenced. The process involved purification of the cDNA fragment using the Qiagen purification kit following the manufacturer’s protocol, cycle sequencing with the HIV-1 genotyping kit with integrase (CDC Atlanta) following the CDC protocol, and analysis using an ABI Prism 3500 Genetic Analyzer (Life Technologies, Waltham, MA, USA). A customized RECall_version 2.28 software was used to edit the raw sequence data and generate consensus sequences, followed by DRM analysis using the Stanford HIVdb program. The phylogenetic analysis was performed using the British Columbia Centre for Excellence (BCCfE) in HIV/AIDS HIVDR quality control (QC) Tool (https://recall.bccfe.ca/whoqc/, accessed on 13 February 2024) [17], neighbor-joining phylogenetic trees were constructed using the molecular evolutionary genetics analysis tool (MEGA versions 6.0 and 11) software, drug-resistance analysis was performed using the Stanford Calibrated Population Resistance (CPR) tool, and the HIVdb web program was utilized to evaluate the sequences obtained for any instances of cross-contamination and quality control.

#### 2.2.3. Next-Generation Sequencing

Following the manufacturer’s instructions, the amplified PCR products of the HIV INT region were cleaned using the Qiagen purification kit (Qiagen, Hilden, Germany), and 10 µL of the cleaned PCR amplicons were used for quantification using the Qubit fluorometer (Invitrogen Thermo-scientific, Waltham, MA, USA) and the qubit ds DNA HS assay kit. PCR products were diluted to 0.2 ng/µL, and then sequencing libraries were prepared using the Nextera XT DNA library preparation kit (Illumina, San Diego, CA, USA) according to the manufacturer’s protocol [18]. Library preparation included fragmentation based on transposon technology and a PCR step incorporating dual-indexes to the fragments and simultaneously tagging the DNA with adaptor sequences. Library normalization was performed for equal library representation during sequencing using the Nextera library normalization kit to obtain a 10–12 pM library. The libraries were diluted and pooled prior to sequencing in a MiSeq. Denatured Phix control (20 pM) from the phix kit was spiked at 20% in the pooled amplicons and was used as a control. Raw MiSeq data obtained in FAST Q format were processed using HyDRA web (https://hydra.canada.ca/, accessed on 13 February 2024) for analysis. HIVDR mutations detected at >1% frequency were reported basing on the default HyDRA Web Mutation Database. Hydra generated reads were reported with estimated frequencies for both minority and high-abundance mutations.

Samples sequenced by both the Sanger and MiSeq-based assays were analyzed for nucleotide and amino acid percent identity using MEGA 6.0. Sequences from this study were deposited in GenBank under accession numbers PX464761-PX464934. DRMs were identified according to the international Antiviral society (IAS) list, and HIVDR was defined as low, intermediate, or high levels and major or accessory according to the HIVdb Stanford algorithm (https://hivdb.stanford.edu/, accessed on 13 February 2024). Data was entered into Epidata and imported to STATA-V18 for analysis.

### 2.3. Statistical Analysis

Continuous variables were summarized using the median (interquartile range) for skewed data. Categorical variables were summarized as proportions and percentages. Viral load was transformed on a log base 10 scale because of the skewed distribution. All statistical analyses were conducted using STATA V.18 (Stata Corp LP, College Station, TX, USA). To evaluate sensitivity, the ratio of positive test results obtained using NGS was utilized while for specificity, the number of negative test results obtained using NGS and Sanger sequencing was divided by the overall number of negative test results obtained from Sanger sequencing. The positive predictive value was determined by dividing the number of test results that were positive in both NGS and Sanger sequencing by the total number of tests that were positive in NGS, while the negative predictive value was determined by dividing the number of test results that were negative in both NGS and Sanger sequencing by the total number of tests that were negative in NGS. Accuracy was defined as the overall probability that a participant’s sample would be correctly classified.

To evaluate the prevalence of DR, the number of study participants with major DRMs was divided by the total number of all study participants. R software version 4.3.1 was used for graphs for DR patterns. To determine the factors associated with the presence of INSTIs DRMs, odds ratios (ORs) were computed using a logistic regression model, and data was assessed for outliers and collinearity. All independent variables which had *p*-values less than 0.2 when examined for their association with the dependent variable at bivariate analysis, and those known from the literature that were associated or were confounders were considered for multivariable analysis. Independent variables were considered statistically significant at 95% confidence interval and *p*-value < 0.05.

### 2.4. Ethical Considerations

Ethical approval was obtained from School of Medicine Research Ethics Committee (SOMREC) (Ref number: Mak-SOMREC-2023-595). The UVRI Research and Ethics Committee (UVRI-REC) granted administrative clearance. A waiver of informed consent was obtained from SOMREC for use of existing medical records. Data was kept confidential, and unique identification numbers were assigned to the participants.

## 3. Results

### 3.1. Study Demographics, Medical History, and Clinical Characteristics

A total of 253 plasma samples of people living with HIV were enrolled during the inclusion period, and, of these, 175 were successfully genotyped and sequenced.

#### 3.1.1. Socio Demographic Characteristics of Participants

A total of 175 participant samples were successfully genotyped and analyzed in this study, the majority being female (*n* = 105, 60.0%), with a median age (IQR) of 36 (27,46) years. A relatively high number of participants obtained their drug refills from HCIII (*n* = 49, 28.0%) followed by HCIV (*n* = 33, 18.9%). Most of the participants were from the Northern part of Uganda (*n* = 59, 33.7%), followed by the Central region (*n* = 54, 30.9%) (see Table 1).

#### 3.1.2. Clinical and Medical History Characteristics of Participants

Regarding treatment, most of the participants were on ART for less than two years (*n* = 68, 38.9%), and there was a high proportion (*n* = 82, 46.9%) on INSTIs (Dolutegravir) for at least two years. A significant number of participants were on TDF/3TC/DTG regimen. Additionally, a relatively high proportion of participants had subtype A (*n* = 121, 69.1%) and D (*n* = 46, 26.3%) in the amplified integrase region. Many of the participants adhered to ART (*n* = 116, 66.3%) and had median log10viral load of 4.2479 (see Table 2).

### 3.2. Diagnostic Accuracy of NGS

#### 3.2.1. INSTI Major DRMs

Using the Illumina MiSEQ platform, NGS accurately detected all DRMs (*n* = 7) at high frequencies (≥20%) that were identified by Sanger sequencing (see Table 3), resulting in a sensitivity and positive predictive value of 100%. NGS did not identify any additional major DRMs at high frequencies (≥20%) (see Table 3), but rather detected additional major DRMs at low frequencies (<20% of the viral population), leading to a specificity and negative predictive value of 100% (Table 4). The overall accuracy of NGS was determined to be 100%. The detailed findings are presented in the tables below.

#### 3.2.2. INSTI Accessory Mutations

NGS accurately detected all of the accessory mutations identified by Sanger sequencing; however, it detected additional mutations that were not detected by Sanger at high frequencies (≥20%) (see Table 5), resulting in a sensitivity of 80% (95%, CI: 61.4–92.3%) and a negative predictive value of 96% (95%, CI: 91.6–98.5%). NGS did not identify participants with accessory mutations as negative at high frequencies (≥20%) (Table 5), hence a specificity of 100% (95%, CI: 97.5–100.0%) and positive predictive value of 100% (95%, CI: 85.8–100.0%) (Table 6). The overall accuracy of NGS was determined to be 96.6% for the detection of accessory mutations. The detailed findings are presented in the tables below.

### 3.3. Prevalence of Drug Resistance Detected by NGS Among Adults Living with HIV on INSTIs with Virological Failure

According to the data presented in Table 7, the overall prevalence of virological failures with major INSTI-associated drug-resistant mutations (DRMs) detected by next-generation sequencing (NGS) at ≥20% threshold/frequency was 4% (95% CI: 1.6–8.0%).

There was an inverse correlation between the prevalence of DRMs and the detection thresholds. The prevalence at different thresholds detected using NGS were 8% (95% CI: 4.4–13.1) at ≥10% threshold, 18.3% (95% CI: 12.9–24.8) at ≥5% threshold, 53.7% (95% CI: 46.0–61.3) at ≥2% threshold, and 60% (95% CI: 53.1–67.6) at ≥1% threshold.

#### 3.3.1. Patterns of Major Mutations Detected by NGS at ≥1% Threshold

**Figure 1:**
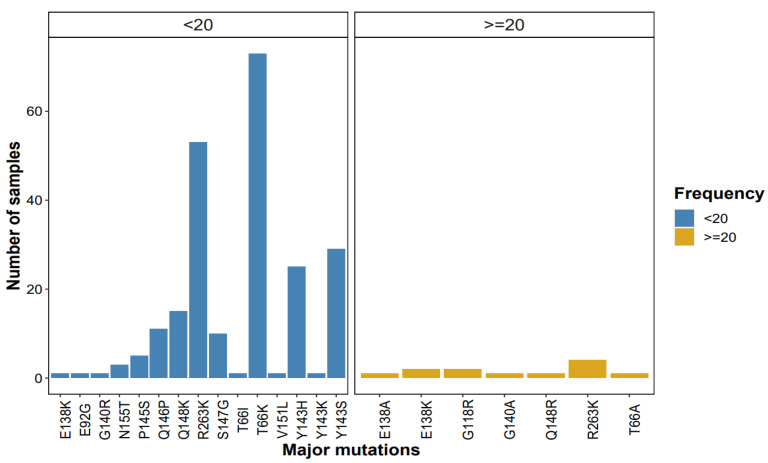
Patterns of major mutations detected by NGS at <20% (blue bars) and ≥20% (yellow bars) frequency/threshold.

Among the samples with major mutations, the predominant ones observed at high frequencies (≥20%) were R263K (*n* = 4/7) followed by G118R (*n* = 2/7), and E138K (*n* = 2/7). Additionally, the major mutations detected at lower frequencies (<20%) were mostly T66IK followed by Y143HKS, R263K, Q148KR, and Q146P. Notably, the major mutations exclusively detected at low-frequency thresholds and not detected at high frequencies were E92G, F121CY, and N155T (see Figure 1 below).

Accessory INSTI-associated mutations were more common, occurring in 19% (*n* = 34) of participants at ≥20 threshold.

#### 3.3.2. Patterns of Accessory Mutations Detected by NGS at ≥1% Threshold

**Figure 2:**
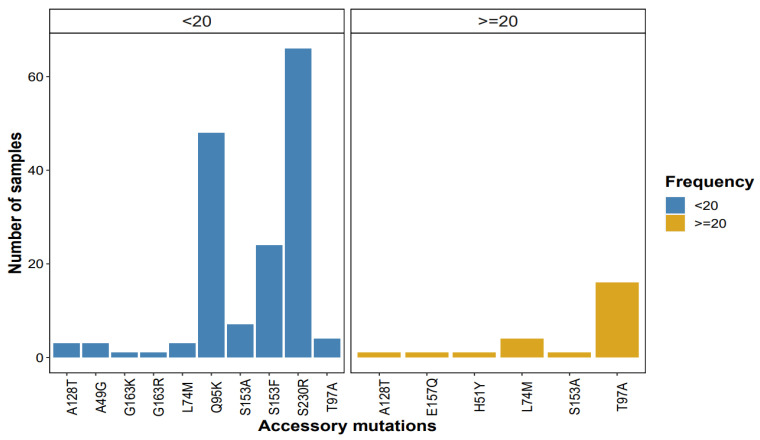
Patterns of accessory mutations detected by NGS at <20% (blue bars) and ≥20% (yellow bars) frequency/threshold.

The accessory mutations that were predominant at high frequencies were T97A and L74M, while at low-frequency thresholds, the predominant mutations were S230R, Q95K, and S153AFY (see Figure 2 below).

### 3.4. Factors Associated with HIVDR Among Adults on INSTIs with Virologic Failure at UVRI

#### Bivariate Analysis of Factors Associated with Presence of DRMs Among 175 Virologic Failures on INSTIs

In the bivariate analysis, each variable was tested independently for the association with the presence of any DRMs; there was no variable with a *p*-value less than 0.2 to be considered for multivariable analysis (see Table 8). Age, sex, duration on ART, current regimen, viral load, and subtype were considered for multivariable analysis, since they are significantly associated with DR according to the literature. However, no variable was deemed statistically significant enough with the presence of DRMs to proceed with multivariable analysis.

## 4. Discussion

### 4.1. Diagnostic Accuracy of NGS Compared to Sanger Sequencing

In this study, samples of participants with major mutations detected by Sanger sequencing and NGS at a high frequency ≥20% of the viral population, mirroring the gold standard in determining diagnostic accuracy, were analyzed. NGS successfully detected all of the mutations detected in SS, suggesting robustness in the detection of DRMs; there were neither false positives nor false negatives detected. Thus, the overall accuracy of NGS was 100%, with sensitivity being 100% (CI: 59.0–100.0), specificity being 100% (CI: 97.8–100.0), negative predictive value being 100% (CI: 97.8–100.0), and positive predictive value being 100% (CI: 59.0–100.0).

The high sensitivity observed in this study is similar to what was reported in a study by Parkin et al., where a 99.9% sensitivity was reported at the 20% threshold [19]. Specificity was equally high (99.78%) at high frequencies in a study conducted in Canada and the USA [20]. The negative and positive predictive values in this study concur with other studies at ≥20 frequency [21,22,23].

The prevalence of major mutations at high abundance was 4% in this study. These findings mirror those reported in the surveys conducted in Malawi, Uganda, and Ukraine that found levels of resistance among those receiving dolutegravir-based antiretroviral treatment with viral non-suppression ranging from 3.9% to 8.6% detected by Sanger sequencing [24].

The study’s overall accuracy was 100%, and aligns with findings from a study conducted in a similar setting that reported approximately a similar accuracy of 98% for the NGS-based assay compared to the Sanger sequencing platform [25]. There is no specific study that was found to have compared Sanger sequencing to NGS at the low-frequency threshold.

The results of this study align with those of other studies, demonstrating agreement between next-generation sequencing (NGS) and Sanger sequencing in detecting the major mutations that are highly abundant. However, the small number of true positives for major DRMs (*n* = 7) influences the precision of our diagnostic accuracy estimates, and the wide confidence intervals for sensitivity (59–100%) reflect the statistical uncertainty inherent to the limited sample size. NGS had a high accuracy due to its deeper sequencing depth resulting from parallel sequencing, unlike the Sanger sequencing platform [25,26].

NGS accurately detected all of the accessory mutations identified by Sanger sequencing and additionally identified accessory mutations that were not detected by Sanger but that were present at high frequencies (≥20%). This resulted in a sensitivity of 80% (95% CI: 61.4–92.3%) and a negative predictive value of 96% (95% CI: 91.6–98.5%). NGS did not falsely classify participants with accessory mutations as negative at high frequencies (≥20%), leading to a specificity of 100% (95% CI: 97.5–100.0%) and a positive predictive value of 100% (95% CI: 85.8–100.0%). The overall accuracy of NGS in detecting accessory mutations was found to be 96.6%. NGS exhibited high concordance with Sanger sequencing; however, confirmation of these diagnostic accuracy estimates in larger and diverse cohorts is necessary to ensure their robustness and generalizability. Given that Sanger sequencing is the gold standard for HIVDR testing in Uganda, the results provide evidence that drug-resistance detection by NGS is effective and detects additional minority variants that confer reduced drug susceptibility, increasing the drug-resistance mutation profile for people on INSTIs; thus, potential healthcare cost reductions can be obtained using early diagnosis and personalized treatments. This suggests that NGS can be used as a screening test for cases with virologic failure that have good ART adherence to guide physicians on the choice of treatment regimens at clinical level and inform policy on consolidated treatment guidelines and resistance-control strategies at surveillance level.

### 4.2. Prevalence of DRMs Detected by NGS (≥20%) Among Adults on INSTIs with VF

In this cross-sectional study, the prevalence of adults with major INSTI associated DRMs was 4% [95% CI: 1.6–8.0], detected at ≥20% threshold. Similar findings reported by surveys conducted in Malawi, Uganda, and Ukraine show levels of resistance among those receiving dolutegravir-based antiretroviral treatment with viral non-suppression ranging from 3.9% to 8.6% detected by Sanger sequencing [24]. However, our findings were lower compared to the 47% detected by NGS reported in a study by Ndashimye et al. [13]. This discrepancy may be due to differences in the populations studied in Ndashimye et al.’s research which was conducted among people on third-line therapy, who are more prone to drug resistance from ART selection pressure [13]. The overall prevalence implies that 1 in every 25 adults with VF on DTG-based ART could have a considerable burden of drug resistance, and this needs to be given due attention, especially the minority variants (low abundance), as they are undetectable by conventional Sanger sequencing and have the potential to merge as the dominant viral population. These findings are consistent with numerous other studies that demonstrate the effectiveness of NGS in identifying high-abundance mutations in addition to low-frequency HIV-drug-resistance mutations [27,28,29,30].

Major mutations, T66K, E92Q, E138K, Q148RK, R263K, and G118R, were detected more often in this study among ART-experienced adults on INSTIs with VF while receiving a DTG-containing regimen, and this finding mirrors results from another study of clinical trials [31]. These mutations independently confer intermediate-to-high-level dolutegravir resistance. In other words, without being combined with other mutations, these specific mutations can reduce the susceptibility to DTG, raltegravir (RAL), and bictegravir (BIC) by 2 fold to 10. This helps to explain why PLHIV harboring these mutations may experience virologic failure or cross resistance [32,33,34].

Mutations at integrase codons 138, 143, 140, 148, and 155, together with other mutations, are known to confer INSTI resistance, and these were detected in a significant number of PLHIV on DTG regimens in this study; similar findings were observed in other studies [27]. Q148H/K/R, in combination with G104S/A/C or E138K/A, reduces DTG susceptibility by two- to ten-fold. Furthermore, the combination of Q148H/R/K + (E138K ± G140S/A) + one additional mutation such as N155H or the accessory mutations L74M and T97A reduce DTG susceptibility >ten-fold [35]. N155H in combination with other INSTI-associated DRMs contributes reduced susceptibility to DTG and possibly BIC. Several mutations are required in HIV integrase to confer high-level resistance to dolutegravir. Cross-resistance studies with raltegravir- and elvitegravir-resistant viruses indicate that Q148H/R and G140S in combination with mutations L74I/M, E92Q, T97A, E138A/K, G140A, or N155H are associated with 5–20-fold reduced dolutegravir susceptibility and virologic failure in patients [33,36].

The accessory mutations S230R, E157Q, L74F, T97A, and S153YAF observed in this study have minimal effects on DTG susceptibility when independent, but, in combination with other major resistance mutations, they synergistically reduce susceptibility to DTG [37,38].

In this study, various DRMs were detected in HIV samples using next-generation sequencing at low abundance, which were notdetected by conventional Sanger sequencing. Notably, mutations such as T66IK, Y143HKS, R263K, Q148KR, Q146P, N155T, E92G, and F121CY were identified. Although the clinical relevance of low-abundance variants has not been established for all drug classes, especially variants (<5%), frequency limits for each mutation are not known, which may affect drug susceptibility. It is crucial to note that low-abundance variants have the potential to rapidly replicate and emerge as the dominant viral population due to the selective pressure exerted by antiretroviral therapy (ART). Consequently, this may lead to virologic failure and increased pre-treatment drug resistance; thus, emphasis should be placed on HIVDR testing before ART initiation [30,39,40]. Rising INSTI HIVDR levels threaten UNAIDS 95-95-95 goals to control the HIV epidemic worldwide.

The prevalence of DRMs was inversely correlated with the mutation detection thresholds (frequency), as the overall prevalence of DRMs was 4% at the ≥20% threshold, 8% at the ≥10% threshold, 18.3% at ≥5% threshold, and 53.7% at ≥2% threshold. As the detection threshold decreases, the prevalence of DRMs increases. This shows that Sanger sequencing misses a high proportion of DRMs, increasing the drug-resistance magnitude. These results are similar to other findings from a systematic review on 39 studies [41]. The detection thresholds for accurately identifying low-abundance variants are distinct from the clinically relevant thresholds for low-abundance DRMs. These clinically relevant thresholds may vary depending on factors such as the drug class, specific drug, or the mutation. Such findings have significant implications for both individual- and population-level HIV treatment strategies [21,42].

Further research should be conducted to understand the interpretation of data for clinically relevant thresholds/frequencies of minority variants. This will provide evidence on where such thresholds lie and how they can be most effectively applied, and this characterization will facilitate the optimal use of ART therapies and the better utilization of some potential advantages of NGS, like better sensitivity for LAV detection and de-convolution of complex mixtures. Importantly, bioinformatics thresholds and standardized reporting guidelines for NGS-based HIV-drug-resistance testing are still evolving, with initiatives such as the WHO working toward greater harmonization and consistency across studies [43].

### 4.3. Factors Associated with Drug Resistance Among PLHIV on INSTIs

None of the variables were statistically significantly associated with the presence of major INSTI DRMs, and this may be due to the small sample size as a result of the high failure rate at genotyping, and hence the wider confidence intervals.

A high proportion of females showed DRMs compared to males, although no statistical association between sex and DRMs was found in this study. This result aligns with similar findings in sub-Saharan Africa [29,44]. The literature underscores women’s vulnerability to HIV due to social and biological factors, while men often possess greater sexual power. Gender inequality and discrimination deprive women of rights like education and healthcare, leading to disempowerment and higher infection rates, with delayed HIV care resulting in poorer treatment outcomes.

Geographic region and health facility were not statistically significantly associated with INSTI DRMs in this study. This is contrary to findings from a study in Kenya, where health facility was significantly associated with DRMs [45]. This could be explained by the difference in populations between the two studies, as, in the Kenyan study, it was conducted among children and adolescents in the southern rift valley, and this population faces unique challenges, including poorer treatment outcomes due to drug stock outs, risk for drug-resistance mutations (HIVDRMs), and limited drug formulations.

In our study, adherence was not significantly associated with INSTI DRMs. This could be explained by the individual differences in pharmacokinetics and pharmacodynamics, which can affect drug levels and the development of resistance independently of adherence. There are studies that report findings similar to our study [29]; however, some studies show that medication adherence <90% or unknown adherence status was associated with DRMs [46].

Age was not significantly associated with INSTI DRMs in our study. This may be a result of reporting bias from participants in the primary study, since we used secondary data acquired based on self-reports. There could be a possibility that patients, especially females, report their age to be less than their actual age to avoid being judged by the health workers attending to them. There are studies that report findings similar to our study [29]. However, a study conducted among sub-Saharan countries showed that being an adult aged 15–24 years was associated with high risk of HIV infection in 2020 in sub-Saharan Africa [44].

Viral load and duration on ART or DTG were not significantly associated with DRMs. However, other previous studies differed from our findings, reporting an association between viral load or duration on ART and DRMs [46,47]. These inconsistent results may be due to different treatment regimens, as the effectiveness of an ART regimen relies on the efficacy of the individual ARV drugs within it. When mutations conferring resistance to two of the antiretroviral (ARV) drugs within a triple-drug combination occur, the ART regimen might fail to adequately suppress HIV-1 replication, the different HIV subtypes (as various HIV subtypes have different resilience), differences in patients’ drug metabolism (for the drug to stay in the body), HIV replication capacity, or medication adherence.

The impact of HIV subtype on drug resistance has garnered attention due to the global intensification of ART. Our research reveals that subtype A (70%) was predominant, followed by subtype D. However, our study finds no significant association between subtype and presence of DRMs. Our findings are similar to findings from previous studies [24,47,48]. In contrast, other studies, such as Wainberg et al., have reported conflicting results [49]. This discrepancy may be attributed to differences in populations, particularly among PLHIV on INSTIs, where populations have different predominant HIV subtypes.

Regimen classification and current regimen had perfect predictions, and were thus removed from the analysis. Adherence was removed from the analysis, since there was missing data for more than 30%.

Specimens were collected from across every region of the country and from a wide variety of health facilities (in terms of geography, level, specialization, ownership, etc.); the results are likely representative at the national level. This broad and varied sampling helps to reduce the selection bias that might have occurred if only a few clinics were included. We acknowledge the limitation that possible bias could have been introduced due to the error-prone reverse-transcription PCR step and sequencing error, thus resulting in a high failure rate during genotyping.

## 5. Conclusions

NGS has good overall accuracy in the detection of high-abundance variants in addition to the low-abundance variants not detected by Sanger sequencing and identifies more participants with accessory mutations that are not detected by Sanger at high frequency. The proportion of participants with major INSTI-associated drug-resistance mutations detected by NGS among adults living with HIV on INSTIs with virological failure was 4%.

Scaling up the use of NGS, a robust and deep sequencing platform, in the detection of potential high-abundance variants to improve drug-resistance-mutation profiling and drug-resistance testing before ART initiation is recommended, as these DRMs can be transmitted to ART-naïve PLHIV, increasing the DR magnitude.

## Figures and Tables

**Table 1:** Socio-demographic characteristics among 175 adults living with HIV with VF at MRC from Jan 2022 to Dec 2022.

Variable	Category	Median (Q1, Q3)	All *n* (%)
Sex	Female		105 (60.0)
	Male		70 (40.0)
Age		36 (27, 46)	
Health facility	HCII		3 (1.7)
	HCIII		49 (28.0)
	HCIV		33 (18.9)
	General hospital		30 (17.1)
	Regional referral		29 (16.6)
	Other		31 (17.7)
Region	Central		54 (30.9)
	Northern		59 (33.7)
	Eastern		26 (14.9)
	Western		36 (20.6)

(Q1, Q3)—interquartile range.

**Table 2:** Clinical and medical history characteristics among 175 adults living with HIV with VF at MRC from Jan 2022 to Dec 2022.

Variable	Category	Median (IQR)	All *n* (%)
Duration on ART in year(s)	1–2		68 (38.9)
	2–5		45 (25.7)
	>5		62 (35.4)
Duration on INSTIs in year(s)	1		41 (23.4)
	2		82 (46.9)
	3		36 (20.6)
	4		16 (9.1)
Current ART regimen	TDF/3TC/DTG		170 (97.1)
	ABC/3TC/DTG		4 (2.3)
	AZT/3TC/DTG		1 (0.6)
Log10Viral load copies/mL		4.248 (3.568, 4.959)	
HIV subtype	A1		121 (69.1)
	D		46 (26.3)
	C		6 (3.4)
	B		1 (0.6)
	Unique recombinant		1 (0.6)
Adherence	Good >95%		116 (66.3)
	unknown		59 (33.7)

(IQR)—interquartile range, TDF—Tenofivir,3TC-Lamivudine, DTG—Dolutegravir, ABC—Abacavir, AZT—Zidovudine.

**Table 3:** Comparison of next-generation sequencing with Sanger sequencing in the detection of major DRMs.

	Sanger Sequencing			
NGS (≥20%)		Positive	Negative	
	positive	7	0	7
	negative	0	168	168
	total	7	168	175

**Table 4:** Validity of next-generation sequencing compared to Sanger sequencing for major mutations.

	Proportion (%)	95% CI
Prevalence	4%	1.6–8.1
Sensitivity	100%	59.0–100
Specificity	100%	97.8–100
Positive Predictive Value	100%	59.0–100
Negative Predictive Value	100%	97.8–100
Overall Accuracy	100%	
Positive Likelihood Ratio	N.S	
Negative Likelihood Ratio	0	

N.S—not significant.

**Table 5:** Comparison of next-generation sequencing with Sanger sequencing in the detection of accessory mutations.

	Sanger Sequencing			
NGS (≥20%)		Positive	Negative	
	positive	24	6	30
	negative	0	145	145
	total	24	151	175

**Table 6:** Validity of next-generation sequencing compared to Sanger sequencing for accessory mutations.

	Proportion (%)	95% CI
Prevalence	17.10%	11.9–23.6%
Sensitivity	80.00%	61.4–92.3%
Specificity	100.00%	97.5–100.0%
Positive Predictive Value	100.00%	85.8–100.0%
Negative Predictive Value	96.00%	91.6–98.5%
Overall Accuracy	96.60%	
Positive Likelihood Ratio	N.S	
Negative Likelihood Ratio	0.2	0.10–0.41

N.S—not significant.

**Table 7:** Prevalence of major DRMs at various thresholds detected by NGS among 175 adults on INSTIs with virologic failure.

NGS Threshold	N (%)	95% CI
≥20%	7 (4.0)	1.6–8.0
≥10%	14 (8.0)	4.4–13.1
≥5%	32 (18.3)	12.9–24.8
≥2%	94 (53.7)	46.0–61.3
≥1%	105 (60.0)	53.1–67.6

**Table 8:** Bivariate analysis for socio-demographic, clinical and medical factors associated with presence of INSTI DRMs among 175 adults with virologic failure.

Variable	DRMs		OR	95% CI	*p*-Value
	Yes	No			
**Sex**					
Female	3 (2.9)	102 (97.1)	Reference		
Male	4 (5.7)	66 (94.3)	2.061	0.447–9.503	0.354
**Age**					
18–26	1 (2.9)	33 (97.1)	Reference		
27–44	3 (3.2)	92 (96.8)	1.076	0.108–10.710	0.950
≥45	3 (6.5)	43 (93.5)	2.302	0.228–23.152	0.479
**Health facility**					
Health center *	5 (5.9)	80 (94.1)	Reference		
Hospital *	1 (1.7)	58 (98.3)	0.276	0.031–2.424	0.246
TASO	1 (3.2)	30 (96.8)	0.533	0.059–4.754	0.573
**Region**					
Central	1 (1.9)	53 (98.1)	0.259	0.028–2.397	0.234
Northern	4 (6.8)	55 (93.2)	Reference		
Eastern	1 (3.8)	25 (96.2)	0.550	0.058–5.175	0.601
Western	1 (2.8)	35 (97.2)	0.393	0.042–3.660	0.412
**Duration on ART in year(s)**					
1–2	1 (1.5)	67 (98.5)	Reference		
2–5	3 (6.7)	42 (93.3)	4.785	0.481–47.534	0.181
>5	3 (4.8)	59 (95.2)	3.406	0.345–33.644	0.294
**Duration on INSTIs in year(s)**					
1–2	4 (3.4)	133 (96.6)	Reference		
>2	3 (5.17)	55 (94.8)	1.541	0.333–7.125	0.580
**Viral load**			0.643	0.233–1.774	0.394
**HIV subtype**					
A1	5 (4.1)	116 (95.9)	Reference		
Others *	2 (3.7)	52 (96.3)	0.892	0.168–4.750	0.894

OR—odds ratio, CI—confidence interval, health center *—(HCII-3, HCIII-49, HCIV-33), hospital *—(general hospital—30, regional referral—29); HIV subtype B (1/175), subtype C (6/175) subtype D (46/175), and unique recombinant (1/175). * Symbolizes categories not mentioned in the table, but defined at the end of the table.

## Data Availability

The datasets generated and analyzed during the current study are not publicly available due to the restriction and data protection policies of the MRC/UVRI & LSHTM, but can be made available by the corresponding authors on reasonable request and on approval of the UVRI Research Ethics Committee.
